# Pan-Cancer Prediction of Cell-Line Drug Sensitivity Using Network-Based Methods

**DOI:** 10.3390/ijms23031074

**Published:** 2022-01-19

**Authors:** Maryam Pouryahya, Jung Hun Oh, James C. Mathews, Zehor Belkhatir, Caroline Moosmüller, Joseph O. Deasy, Allen R. Tannenbaum

**Affiliations:** 1 Department of Medical Physics, Memorial Sloan Kettering Cancer Center, New York, NY 10065, USA; maryam.pouryahya@gmail.com (M.P.); mathewj2@mskcc.org (J.C.M.); deasyj@mskcc.org (J.O.D.); 2 School of Engineering and Sustainable Development, De Montfort University, Leicester LE1 9BH, UK; zehor.belkhatir@dmu.ac.uk; 3 Department of Mathematics, University of California at San Diego, La Jolla, CA 92093, USA; cmoosmueller@ucsd.edu; 4 Departments of Computer Science and Applied Mathematics & Statistics, Stony Brook University, Stony Brook, NY 11794, USA; allen.tannenbaum@stonybrook.edu

**Keywords:** drug sensitivity, optimal mass transport, network-based clustering, cell lines

## Abstract

The development of reliable predictive models for individual cancer cell lines to identify an optimal cancer drug is a crucial step to accelerate personalized medicine, but vast differences in cancer cell lines and drug characteristics make it quite challenging to develop predictive models that result in high predictive power and explain the similarity of cell lines or drugs. Our study proposes a novel network-based methodology that breaks the problem into smaller, more interpretable problems to improve the predictive power of anti-cancer drug responses in cell lines. For the drug-sensitivity study, we used the GDSC database for 915 cell lines and 200 drugs. The theory of optimal mass transport was first used to separately cluster cell lines and drugs, using gene-expression profiles and extensive cheminformatic drug features, represented in a form of data networks. To predict cell-line specific drug responses, random forest regression modeling was separately performed for each cell-line drug cluster pair. Post-modeling biological analysis was further performed to identify potential biological correlates associated with drug responses. The network-based clustering method resulted in 30 distinct cell-line drug cluster pairs. Predictive modeling on each cell-line-drug cluster outperformed alternative computational methods in predicting drug responses. We found that among the four drugs top-ranked with respect to prediction performance, three targeted the PI3K/mTOR signaling pathway. Predictive modeling on clustered subsets of cell lines and drugs improved the prediction accuracy of cell-line specific drug responses. Post-modeling analysis identified plausible biological processes associated with drug responses.

## 1. Introduction

Recent significant advances in investigating drug sensitivity have been driven by advances in high-throughput technologies that can generate large amounts of biological data at low cost. Pioneers of such datasets include the NCI-60 database [1], Genomics of Drug Sensitivity in Cancer (GDSC) project [2], and Cancer Cell Line Encyclopedia (CCLE) project [3]. Collectively, these databases have demonstrated that pharmacogenomic profiling of cancer cell lines from clinical tumor samples can help guide the development of new cancer therapies [4,5]. The NCI-60 project is one of the first established studies for in vitro drug screening, and has significantly improved the philosophy and research of human cancer drugs [1,6]. This panel has led to many important discoveries, including a general advance in understanding the underlying mechanisms of cancer in response to drugs [7,8]. However, the panel only consists of 60 cell lines, which limits its use for developing reliable predictive models. By contrast, the GDSC database (http://www.cancerRxgene.org, accessed on 9 December 2021), on which we focus in this study, annotates a comprehensive landscape of drug responses of ∼1000 human cancer cell lines for 265 anti-cancer drugs. Importantly, the genomic and transcriptomic profiles of all cancer cell lines employed in GDSC were extensively characterized as a part of the COSMIC cell line project (CCLP, https://cancer.sanger.ac.uk, accessed on 9 December 2021). These resources have the potential to link anti-cancer drug sensitivity to detailed genomic information and facilitate the discovery of relevant molecular biomarkers when coupled with powerful analytical tools to cope with the high-dimensionality and complexity of these datasets.

A variety of approaches have been proposed for investigating drug sensitivity in cancer cell lines. One of the first models was developed by Staunton et al., which employed a weighted voting classification model for anti-cancer drug sensitivity based on NCI-60 gene-expression data [9]. Recent approaches can be grouped either as regression models to predict the concentration required for inhibition, or classification prediction models of drug responses as sensitive vs. resistant [10], or a mathematical modeling approach [11]. Machine learning tools deployed include support vector machines [12], random forests [13], neural networks [14], and logistic ridge regression [15]. For example, Riddick et al. built an ensemble regression model with random forest to predict in vitro drug responses using gene-expression profiles [16].

In the present study, we demonstrate that cell-line and drug clustering prior to machine learning modeling can significantly improve the accuracy of cell-line drug-sensitivity prediction. We first represented genomic profiles of cell lines and chemical features of drugs in the form of separate feature networks. Several network-based papers for drug-sensitivity prediction have been previously published. For instance, Wang et al. proposed a heterogeneous network model of cell lines, drugs, and targets [17,18]. Zhang et al. proposed a dual-layer cell-line drug network model for the prediction of drug responses [19]. These studies found that similar cell lines respond very similarly to a given drug, and structurally related drugs also have similar responses to a given cell-line. Stanfield et al. introduced a network-based method for drug-response prediction using a large, heterogeneous network consisting of genes, cell lines, and drugs, where each cell- line and genes with mutations in the given cell-line were linked. Sensitivity and resistance scores were then computed for each cell-line drug pair [20]. Ahmed et al. employed a network-based feature selection method using a gene co-expression network [21]. The resulting output was then used in neural network models for drug-response prediction. Compared to these network-based models, our method has some potential advantages. In particular, we employed machine-learning-based modeling on integrated gene-expression profiles from cell lines together with cheminformatic features from drugs. More importantly, the predictive modeling was performed on more homogeneous subsets after the clustering of 915 cell lines and 200 drugs based on the similarity between them, resulting in improved predictive power. Post-modeling biological analysis identified key biological correlates associated with specific clusters (paired clusters of cell lines and drugs). More specifically, we clustered cell lines using optimal mass transport (OMT) theory applied to gene-expression profiles, as represented by a network from the Human Protein Reference Database (HPRD, http://www.hprd.org, accessed on 9 December 2021). [22]. This resulted in a distance between each pair of cell lines, called the Earth Mover’s Distance (EMD), or the Wasserstein distance [23,24,25]. This distance measures the magnitude of the expression signal that needs to be moved from one expression distribution to the other within the network at the minimum cost. A clustering method was then applied to the resultant distance matrix to group the cell lines. Similarly, this procedure was applied to a network of molecular descriptors of drugs, resulting in a set of clusters of drugs. Random forest regression modeling was then conducted on each paired cluster (a cluster of cell lines and a cluster of drugs). This approach outperformed previously developed network-based methods [19,26]. It was also observed that the Wasserstein distance metric is more powerful in predicting the drug responses than Pearson correlation that is generally used in network-based models.

In summary, the heterogeneity of pan-cancer cell lines and structurally diverse drugs in large-scale pharmacogenomic databases makes prediction of drug sensitivity challenging. In this work, we propose a novel computation method for clustering cell lines and drugs in an unsupervised way, followed by a supervised prediction-modeling of drug responses. Our results demonstrate that modeling on homogeneous data significantly improves the prediction accuracy. Moreover, clustering increases the focus for understanding potential biomarkers and mechanisms of drug sensitivity.

## 2. Results

### 2.1. Clustering of Cell Lines and Drugs

**Figure 1:**
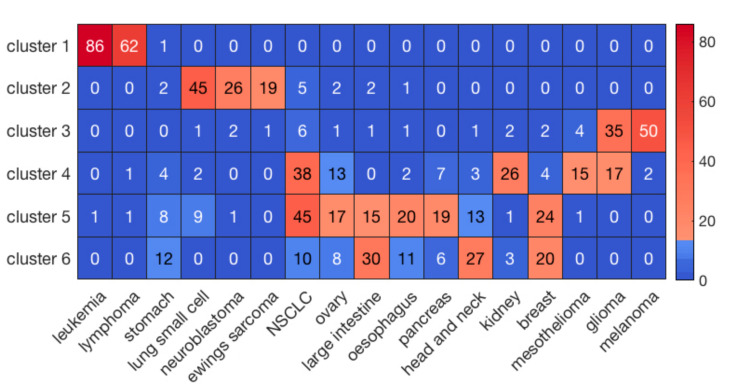
The clustering results of cell lines for the major 17 cancer types. The sidebar indicates the number of cell lines in each element.

Hierarchical clustering of the cell lines resulted in six clusters with the highest average silhouette score. The numbers of cell lines in each cluster were 149, 113, 130, 174, 208, and 141, respectively, labeled clusters 1 through 6. Figure 1 illustrates the results of clustering for 17 major cancer types. As shown in Figure 1, cluster 1 perfectly grouped the liquid cancers of leukemia and lymphoma, including only one solid tumor cell-line. It is well known that liquid tumors respond very differently to anti-cancer drugs compared to solid neoplasms [27]. Interestingly, some clusters, such as cluster 5, consisted of heterogeneous cancer types, perhaps indicating a closer relationship in drug responses. On the other hand, hierarchical clustering of the drugs resulted in 5 clusters. The numbers of drugs in each cluster were 10, 23, 86, 26, and 55, respectively, labeled clusters 1 through 5.

### 2.2. Prediction of Drug Responses in Paired Cell-Line Drug Clusters

**Figure 2:**
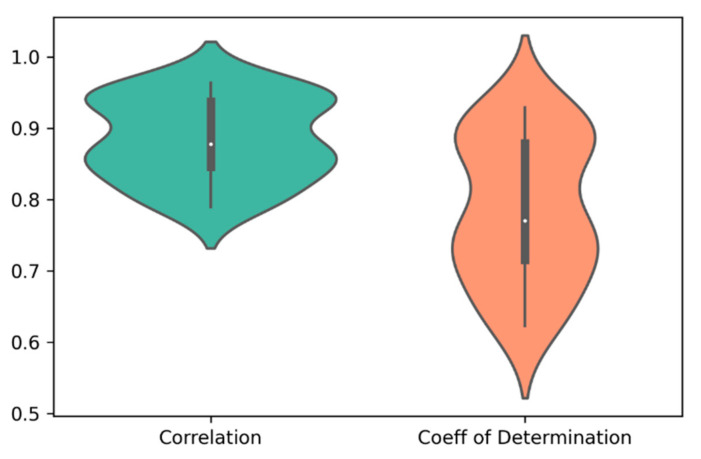
The distribution of correlation (R) and coefficient of determination (R^2^) of the predicted and observed log(IC50) values in the 30 paired clusters of cell lines and drugs. The average values of R and R^2^ were 0.88 and 0.78, respectively.

For each of the 30 paired clusters (six clusters for cell lines and five clusters for drugs), random forest regression models were trained and validated, using 635 genes and 165 cheminformatic features. A three-fold cross-validation approach was employed, such that in each cross validation, 2/3 of the data were used for training, and 1/3 of the data were used for validation of the model. After performing the three-fold cross validation in each paired cluster, correlation (R) and coefficient of determination (R^2^) values were computed for the predicted and observed log(IC50) values. Figure 2 illustrates the distribution of R and R^2^ of the predicted and observed log(IC50) values in the 30 paired clusters of cell lines and drugs.

**Figure 3:**
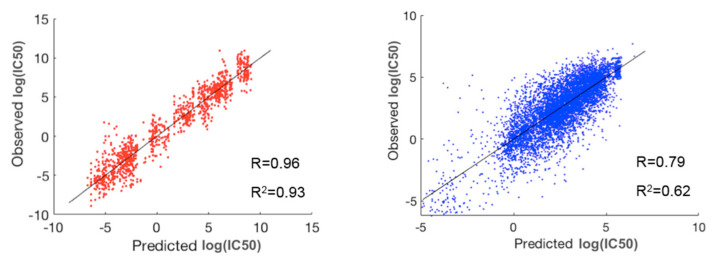
The best (red) and worst (blue) clusters among the 30 paired clusters with respect to prediction accuracy. The best prediction lies in the pair of cell-line cluster 3 (mainly glioma and melanoma) and drug cluster 1. The worst prediction lies in the pair of cell-line cluster 6 (mainly consisting of breast, head and neck, large intestine, and stomach cancers) and drug cluster 5.

**Figure 4:**
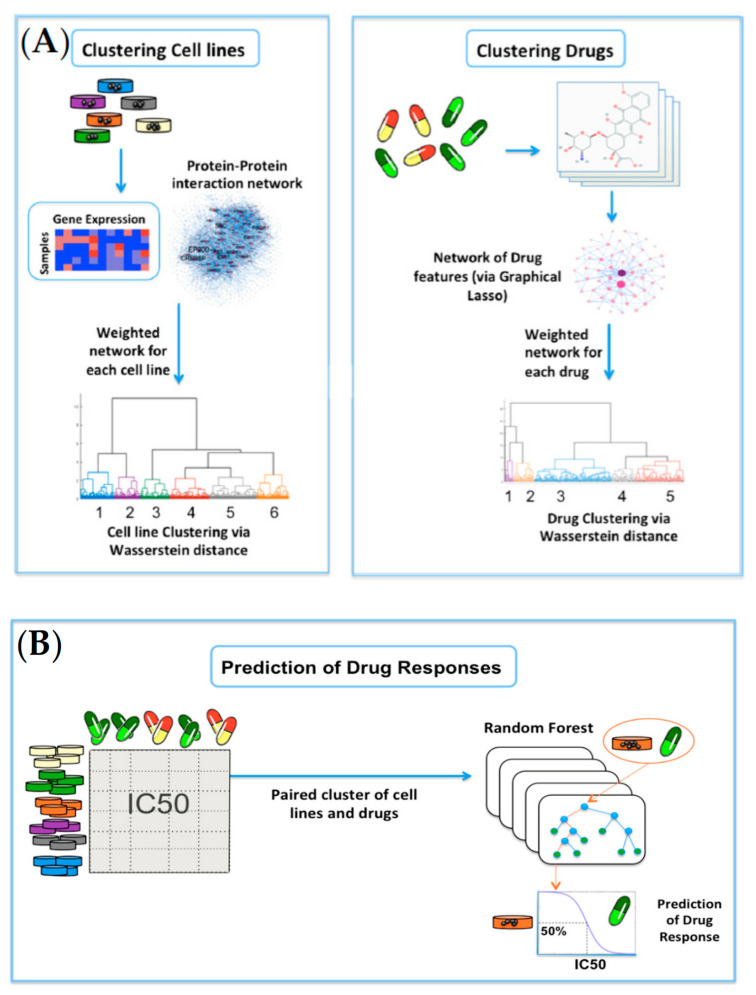
Overview of the network-based clustering and modeling of drug responses: (**A**) For clustering of cell lines, the gene-expression profiles for 915 cell lines were analyzed on the HPRD network. Invariant measures for individual nodes were then computed, and the Wasserstein distance (EMD) was computed between each pair of cell lines on the network. Lastly, hierarchical clustering was performed on the resultant Wasserstein distance matrix. For clustering of drugs, we obtained the cheminformatic features of 200 drugs, and built a data-driven network of cheminformatic features using the graphical LASSO. Similar to cell lines, hierarchical clustering was performed on the resultant Wasserstein distance matrix; (**B**) A random forest model was built on each paired cluster of cell lines and drugs to predict drug responses in log(IC50) values.

To evaluate the performance of prediction for the whole dataset, we concatenated the predicted and observed log(IC50) values for all the 30 clusters and then calculated R and R^2^ (Table 1). For comparison, we also performed a three-fold cross validation scheme via random forest on the whole dataset without prior clustering. As shown in Table 1, our method using prior clustering of cell lines and drugs resulted in prediction accuracies of R = 0.89 and R^2^ = 0.79, outperforming the modeling results (R = 0.77 and R^2^ = 0.60) obtained via random forest on the whole dataset (183,000 cell-line drug pairs) using a three-fold cross validation scheme. Further, R and R^2^ in the best and worst paired clusters with respect to prediction accuracies were (R = 0.96 and R^2^ = 0.93) and (R = 0.79 and R^2^ = 0.62), respectively (Figure 3). The cell-line cluster 3 and drug cluster 1 pair, shown in Figure 4A, achieved the best accuracy. This cluster mainly consisted of glioma and melanoma (Figure 1). In addition, the cell-line drug complex network (CDCN) model coupled with the Wasserstein distance outperformed the model using Pearson correlation.

**Figure 5:**
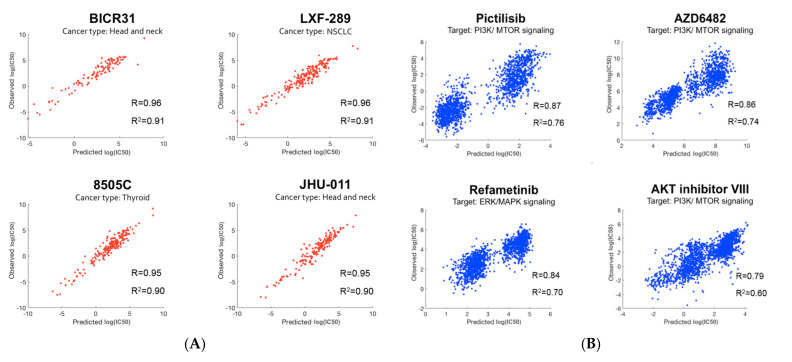
Prediction performance: (**A**) The top four cell lines with the best prediction performance. Cell-line names along with their cancer types are shown. Three out of the top four cell lines belong to head and neck (including thyroid) cancer; (**B**) The top four drugs with the best prediction performance. Drug names along with their targeted pathways are shown. Three out of the top four drugs target the PI3K/mTOR signaling pathway.

After applying the modeling pipeline, we investigated the prediction accuracy for individual cell lines and drugs. Figure 5A,B illustrate prediction performance for the cell lines and drugs with the highest prediction accuracy. As shown in Figure 5A, three of the top four cell lines were from head and neck (including thyroid) cancer. Interestingly, three out of the top four drugs target the PI3K/mTOR signaling pathway, and the remaining one targets the related ERK/MAPK signaling pathway [28].

### 2.3. Biological Analysis

**Figure 6:**
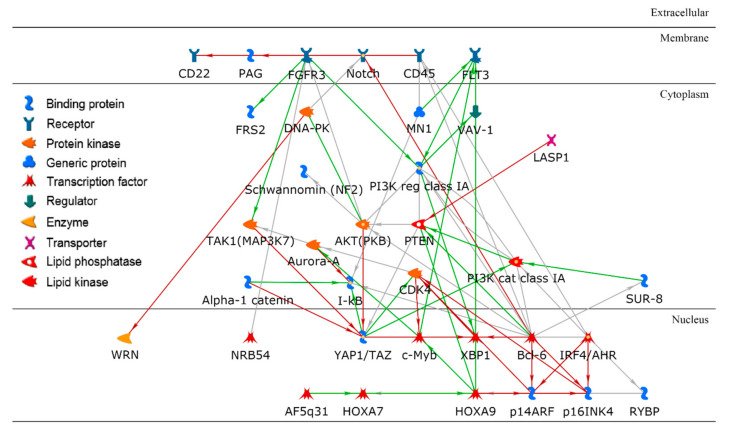
A protein–protein interaction network using a set of key gene products in a paired cluster of cell lines and drugs. Bcl-6 is a hub in the network with the highest node degree.

To identify significant genes, we employed a two-step approach: (1) the importance score for each gene was derived based on its contribution to the random forest accuracy [29] and (2) using a *t*-test, differentially expressed genes were further identified. For example, we investigated a paired cluster: cell-line cluster 4 and drug cluster 1, which is one of the highest performing cluster pairs. Initially, the top 200 genes were selected based on the importance score in random forest modeling, and 70 out of the 200 genes met a Bonferroni corrected *p*-value < 0.05. For these 70 genes, gene ontology enrichment analysis was performed using MetaCore software to discover significant biological correlates. Table 2 shows the top five biological processes, yielding the related processes of apoptosis and programmed cell death as the top two biological processes, with extremely low false discovery rate (FDR) values of 2.55 × 10^−20^. The hypergeometric distribution was used to compute unadjusted *p*-values. For further insight, a protein–protein interaction (PPI) network with direct connections among the set of 70 gene products was constructed as shown in Figure 6.

## 3. Discussion

In this study, we developed a network-based method for predicting the drug sensitivity of pan-cancer cell lines in the GDSC database. Several studies have proposed network-based methods for drug response prediction on single omics data [20,21], whereas the current study used multi-modal genomic and cheminformatic data. The CDCN modeling introduced by Wei et al. [26] and its extended method [19] also used genomic and cheminformatic data [26]. An advantage of our approach compared to the CDCN model is that we employed unsupervised and supervised machine learning methods in connection with OMT theory, demonstrating that random forest modeling in the resulting distinct pairs of cell-line and drug clusters can produce better predictive power. This is in line with a previous study that showed that data preprocessed by a clustering algorithm improved the prediction accuracy of random forest models [30]. We also found that the CDCN model coupled with the Wasserstein distance can improve predictive power compared to the original method using Pearson correlation [18]. In addition, our results indicate that cell lines judged to be similar, according to the Wasserstein distances computed between invariant measures from gene-expression profiles, exhibit similar responses to the (structurally) similar drugs [19,26].

In the application of machine-learning techniques to biology, interpretability is very important. Clustered cell lines and drugs, and the resulting random forest models in individual paired clusters, can be deeply interrogated to gain further insights into the determinants of cell-line drug effectiveness. We have demonstrated that post-modeling analysis using bioinformatics techniques enables the identification of plausible biological correlates. For example, we investigated the pair of cell-line cluster 4 and drug cluster 1 (see Figure 4A). Cell-line cluster 4 consisted mostly of non-small-cell lung cancer (NSCLC), kidney cancer, mesothelioma, and glioma. Drugs in drug cluster 1 have been shown to mainly target mitosis and DNA-replication including antimetabolites. The PPI network, resulting from the set of key genes relevant to the paired cluster, is illustrated in Figure 6. As shown, Bcl-6 (B-cell lymphoma 6) is a hub in the network with the highest node degree. Bcl-6, encoded by the *BCL6* gene, was initially discovered as an oncogene in B-cell lymphomas, driving a malignant phenotype via the repression of DNA damage and proliferation checkpoints [31]. *BCL6* has also been implicated in an expanding spectrum of solid and hematologic tumors, including leukemia, breast cancer, and NSCLC [32]. Additionally, *BCL6* expression has been implicated in the modulation of apoptotic responses of malignant cells to chemotherapeutic reagents, suggesting the development of *BCL6* inhibitors as a potential therapeutic option [33].

A limitation of this study is that using IC50 as the measure of drug sensitivity may be biased, due to different growth rates of cancer cells growing in culture [34]. Moreover, the change in control cell numbers during the observation period can also lead to a bias in IC50 values [35]. Furthermore, we limited our analysis to 635 genes based on the OncoKB database, potentially resulting in a loss of useful information. However, all of the OncoKB genes are known to be related to cancer, and thus highly relevant to this study. Future applications could include organoid or PDX response modeling, which would provide more insights into applicability and anti-cancer drug sensitivity. In the clustering of drugs, highly correlated cheminformatic features were removed in an unsupervised way while keeping non-redundant informative features. Despite this trimming of data, our method achieved better predictive power than other approaches.

## 4. Materials and Methods

### 4.1. Data and Preprocessing

We used the anti-cancer drug-response data from the GDSC database. GDSC is a publicly available large-scale pharmacogenomic database that includes drug-screening data for more than a thousand human pan-cancer cell lines. The dataset consists of 265 compounds, including cytotoxic chemotherapeutics as well as targeted therapeutics. GDSC drug responses are given as log-transformed IC50 values (natural log of drug concentration required to inhibit 50% of growing cells using a proliferation assay) and the area under the curve (AUC) for a fitted model. We used log(IC50) as the degree of drug responses. Genomic mRNA expression profiles (Affymetrix Human Genome U219 Array) of the cell lines within GDSC were obtained from the CCLP database. A protein–protein interaction (PPI) network was obtained from the HPRD database. Cell lines with missing data for more than 80% of the drugs were removed, leaving 915 cell lines (Table S1, Figure S1).

The CCLP and HPRD datasets had 8483 genes in common. Even though our method is applicable to large scale data, in this study, we wanted to focus on genes of known relevance in cancer. Thus, we used a smaller set of genes from the OncoKB (Precision Oncology Knowledge Base) database (http://oncokb.org/, accessed on 9 December 2021) that consists of 1019 genes. Among those 1019 genes, 796 genes were common to both CCLP and HPRD. In the HPRD network with those 796 genes, the largest connected network component consisted of 635 genes, which we focused on in this study (Figure S2). To extract cheminformatic descriptors of drugs, we obtained the chemical structures of the drugs from PubChem (https://pubchem.ncbi.nlm.nih.gov/, accessed on 9 December 2021) and downloaded the SMILES (Simplified Molecular Input Line Entry Specification) string of 241 drugs, for which the PubChem ID was provided in the GDSC database. Two-hundred drugs had response values for more than half of the cell lines, resulting in 183,000 cell-line drug pairs (915 cell lines × 200 drugs; Table S1). We then extracted 1500 cheminformatic descriptors (drug features) of those 200 drugs using Dragon software (version 7.0) by Kode-Chemoinformatics (https://chm.kode-solutions.net/, accessed on 9 December 2021). The descriptors included functional groups, fragment counts, and estimated chemical properties as well as simple atomic descriptors. The overview of data analysis is shown in Figure S2.

### 4.2. The Invariant Measure of Gene Expression in a PPI Network

We have previously found that Markov chain modeling of gene-expression networks results in greatly improved classification. Markov chains model expression levels as a stochastic message-passing process where signals are passed between nodes [36,37]. Hence, we follow a similar approach here. We constructed a weighted graph on the given PPI network as a Markov chain in the following manner. Consider a gene i and its neighboring genes $j\in N\left(i\right)$ in the interaction network (here in HPRD) for a given sample. Let $ge_{i}$ denote the expression level of gene i in a given sample. The principle of mass action implies that the probability $\left(p_{ij}\right)$ of the interaction of gene i to gene j  is proportional to their expressions, i.e., $p_{ij}\propto (ge_{i})(ge_{j})$ [38]. By normalizing $p_{ij}$ so that $\sum_{j}p_{ij}=1$, we can form the stochastic matrix p of the Markov chain associated with the network as follows:

(1) $$p_{ij}:=\frac{ge_{j}}{\sum_{k\in N\left(i\right)}ge_{k}}$$

If we let this stochastic signal-passing process proceed from an initial state based on gene expressions, in repeated steps according to these probabilities (called a Markov chain), it can be shown that the system reaches a stationary distribution, implying that the system is invariant under a right multiplication by p, i.e., πp=π [36]. Solving this formula for the special stochastic matrix p, π has the explicit expression:

(2) $$\pi_{i}=\frac{1}{Z}\left(ge_{i}\times \sum_{j\in N\left(i\right)}ge_{j}\right),$$

where Z is a normalization factor making π a probability vector. Of note, this normalization is necessary since we need the invariant measure to be a probability distribution over all genes for each specific sample. The invariant measure defined by Equation (2) gives a value to each gene which is not only dependent on the gene expression of the gene i, but also on the total gene expressions of the neighboring genes $j\in N\left(i\right)$. For each sample, a vector $\pi =(\pi_{i}){\;}_{i=1,\cdots ,n\;}$ for all the n genes was computed. The Wasserstein distance was then computed to measure the distance between a pair of vectors of the form π assigned to every two cell lines. Lastly, using the resultant Wasserstein distance matrix in a hierarchical agglomerative clustering method, cell lines were clustered, as described below.

### 4.3. Network Construction of Cheminformatic Drug Features via Graphical LASSO

We initially extracted molecular descriptors of the 200 drugs from Dragon software. The following descriptors were removed: descriptors that are constant or near constant and descriptors with missing values, yielding 1500 features. We further removed many highly correlated features via unsupervised clustering, using the Spearman’s correlation between the features. We selected a representative feature from each cluster, which had the highest average correlation to all other intra-cluster features. This further reduced the number of features to 500. We then constructed a network of these cheminformatic features via the graphical LASSO, which suppressed unimportant feature connections to build a sparse network (see the Appendix A for more information about the graphical LASSO) [39,40,41]. The largest connected network component consisted of 165 cheminformatic features. The graphical LASSO method uses regularization to squeeze out less important network edges, while minimizing information loss. We then normalized cheminformatic features on the largest connected network component to sum up to one to be considered a probability distribution. Note that we did not compute the invariant measure used in the PPI network for cell lines, since the data-driven network of cheminformatic features represents the correlation between features rather than the biological interactions. After assigning the resultant network probability distributions to individual drugs, we calculated the Wasserstein distance to measure the similarity between each pair of drugs. Lastly, the resultant Wasserstein distance matrix was input to a hierarchical agglomerative clustering method to cluster drugs.

### 4.4. Network-Based Clustering via the Wasserstein Distance

As described above, cell lines and drugs were separately clustered, using gene-expression profiles and cheminformatic features, respectively, represented in the form of fixed-topology networks. Our network-based clustering method is based on the theory of OMT [23,24,25], employing the *W*_1_ Wasserstein distance (EMD) metric (Figure S3). OMT is a rapidly developing area of research that deals with the geometry of probability densities [23]. The work on OMT was initiated by Gaspard Monge in 1781 [42] who formulated the problem of finding the minimal transportation cost to move a pile of soil to fill an excavation site (see the Appendix B for more information about the Wasserstein distance) [43,44]. Wasserstein distances have unique properties that capture the overall, system-wide differences in data patterns.

The resultant pair-wise Wasserstein distance matrix was input to a hierarchical agglomerative clustering method, resulting in a set of clusters of cell lines or drugs. To find the optimal number of clusters, the silhouette score was used [45]. The silhouette score is a measure used to evaluate the goodness of the number of clusters created by clustering methods. More specifically, for each sample  i, the silhouette score is defined as follows:

$$s\left(i\right):=\frac{b\left(i\right)-a\left(i\right)}{\max\left\{a\left(i\right),b\left(i\right)\right\}}$$

where $a\left(i\right)$ is the average distance of the sample i to all samples within its own cluster, and $b\left(i\right)$ is the minimum average distance of the sample i to samples in a different cluster, minimized over clusters. The optimal number of clusters then has the highest average silhouette score over a range of possible values.

### 4.5. Prediction of Drug Responses in Paired Cell-Line Drug Clusters

Predictive modeling of drug responses was conducted in each cell-line drug cluster, employing random forest regression on the associated gene-expression profiles and cheminformatic features. We chose the number of decision trees to be 100. For all other parameters, the default settings were used: the minimum number of samples in the terminal nodes was set to 5, and the *mtry* parameter was set to *p*/3, where *p* is the number of features [29]. Figure 4 illustrates the pipeline of clustering and random forest modeling. Our method was compared with a cell-line drug complex network (CDCN) model introduced by Wei et al. [26], which is the extension of the dual-layer cell-line drug network model [19]. We assessed the CDCN model with a closed-form formula in each paired cluster of cell lines and drugs, comparing two different metrics of Wasserstein distance and Pearson correlation (see the Appendix C for more information about the CDCN model).

## 5. Conclusions

This study proposed a novel network-based clustering method based on OMT theory for drug response prediction. Clustering was performed for cell lines and drugs using gene-expression profiles and cheminformatic drug features, respectively, represented in the form of data networks. Random forest modeling was then performed for each cell-line drug cluster pair. Prediction modeling on clustered homogeneous data is likely to improve the prediction accuracy for drug sensitivity, as well as enhance the biological interpretability compared to modeling using all the data together. We plan to apply the proposed approach to several biomedical problems with multi-modal data, including genomics and medical imaging.

## Figures and Tables

**Table 1:** Performance comparison of four different models. CDCN: Cell-line drug complex network; WD: Wasserstein distance.

Models	R	R^2^
a. Random forest using prior WD-based clustering	0.89	0.79
b. CDCN model with WD	0.86	0.59
c. Random forest on the whole data	0.77	0.60
d. CDCN model with Pearson correlation	0.74	0.53

**Table 2:** The top five biological processes obtained from gene ontology enrichment analysis using 70 significant genes.

Ranking	Biological Processes	FDR	Number of Input Genes
1	Regulation of apoptotic process	2.55 × 10^−20^	40
2	Regulation of programmed cell death	2.55 × 10^−20^	40
3	Regulation of cell death	4.94 × 10^−20^	41
4	System development	1.93 × 10^−18^	56
5	Positive regulation of nitrogen compound metabolic process	5.35 × 10^−18^	48

## Data Availability

Data used in this experiment are available at https://github.com/mskspi/drugsensitivity/.

## Supplementary Materials

The following supporting information can be downloaded at: https://www.mdpi.com/article/10.3390/ijms23031074/s1.

## Appendix A

**Graphical LASSO**

Consider n observations having a multivariate Gaussian distribution with mean $\mu \;\left(=0\right)$ and covariance matrix Σ. The graphical LASSO is a regularization framework for estimating the covariance matrix S, under the assumption that its inverse (precision matrix), $\Theta =\Sigma^{-1}$, is sparse. If an element $\;\theta_{jk}=0$, this implies that the corresponding variables (vertices) of indices j and k are conditionally independent, given other variables. This can justify removing the edge connecting these two vertices (j and k). The graphical LASSO imposes an $l_{1}$ penalty for the estimation of Σ to increase the graph sparsity. The graphical LASSO problem minimizes an $l_{1}$-regularized negative log-likelihood as follows:

$${\;\mathrm{argmin}}_{\Theta \geq 0}(-\log\;\det\left(\Theta \right)+\mathrm{tr}\left(S\Theta \right)+\lambda {\left||\Theta |\right|}_{1}),$$

where S is the empirical covariance matrix, ${\left||\Theta |\right|}_{1}$ denotes the sum of the absolute values of Θ, and λ is a tuning parameter controlling the amount of $l_{1}$ shrinkage. Here, we tune $l_{1}$ to make the network as sparse as possible, such that it does not decrease the accuracy of drug-response prediction.

## Appendix B

**Wasserstein Distance**

Optimal mass transport theory handles the problem of finding the minimal transportation cost to move a pile of soil, with a mass density $\rho^{0}$, to an excavation site, with a mass density $\rho^{1}$. A relaxed version of the problem was introduced by Leonid Kantorovich in 1942. Let $\rho^{0},\rho^{1}\in P\left(\Omega \right)$ where $\Omega \subseteq {\mathbb{R}}^{N}$ and $P\left(\Omega \right)=\{\rho \left(x\right):\int_{\Omega }^{}\rho \left(x\right)dx=1,\rho \left(x\right)\geq 0\}.$ The *W*_1_ *Wasserstein distance*, also known as the *Earth Mover’s Distance* (EMD), is defined as follows:

$$W_{1}\left(\rho^{0},\rho^{1}\right)=\underset{\gamma \in \Gamma \left(\rho^{0},\rho^{1}\right)}{\inf}\int_{{\mathbb{R}}^{N}\times {\mathbb{R}}^{N}}^{}||x-y||d\gamma \left(x,y\right),$$

where $\Gamma \left(\rho^{0},\rho^{1}\right)$ denotes the set of all couplings between $\rho^{0}$ and $\rho^{1}$, that is, the set of all joint probability measures γ on Ω×Ω whose marginals are $\rho^{0}$ and $\rho^{1}$. Here, the cost function of the transportation is defined as the ground distance $d\left(x,y\right)=||x-y||$.

The optimization problem has an analogous formulation on a weighted graph. Let us consider a connected undirected graph *G* = (*V, E*) with n nodes in *V* and m edges in *E*. Given two probability densities $\rho^{0},\rho^{1}\in$ ${\mathbb{R}}^{n}$ on the graph, the Wasserstein distance problem seeks a joint distribution $\rho \in {\mathbb{R}}^{n\times n}$ with marginals $\rho^{0}$ and $\rho^{1}$ minimizing the total cost $\sum c_{ij}\rho_{ij}$:

$$\;W_{1}\left(\rho^{0},\rho^{1}\right)=\underset{\rho }{\min}\left\{\overset{n}{\underset{i,j=1}{\sum }}c_{ij}\rho_{ij}\;|\;\sum_{k}\rho_{ik}=\rho_{i}^{0},\sum_{k}\rho_{kj}=\rho_{j}^{1},\;\;\forall i,j\right\}.$$

Here $c_{ij}$ is the cost of moving unit mass from node i to node j in the shortest path. Therefore, the Wasserstein distance in our study is a network-based metric that considers the network connectivity in calculating the cost function.

## Appendix C

**Cell-Line Drug Complex Network Model**

Assume that $r\left(c,d\right)$ is a log(IC50) value of a pair of cell-line c∈C and drug d∈D where C and D denote a set of cell lines or drugs, respectively. For a new cell-line $c^{*}$ and a new drug $d^{*}$, we would like to predict the drug response, $r(c^{*},d^{*})$, based on the known values $r\left(c,d\right)$. Using the metric $d_{D}$ (Wasserstein distance), we can cluster $\;D\cup \{d^{*}\}$. Denote by $C_{d^{*}}$ the cluster in which $d^{*}$ lies, but with $d^{*}$ removed from it. Similarly, we can compute $C_{c^{*}}$ in the cell-line space using the $d_{C}$. We define a similarity weight function as $w\left(c,\;c^{*}\right)=e^{-\frac{{\left|d_{C}\left(c,c^{*}\right)\right|}^{2}}{2\alpha^{2}}}$ between cell lines and a similarity weight function between drugs as $w\left(d,\;d^{*}\right)=e^{-\frac{{\left|d_{D}\left(d,d^{*}\right)\right|}^{2}}{2\beta^{2}}}$ with the vector of decay parameters, i.e., $\zeta =\left(\alpha ,\beta \right)$. These similarity weights are defined such that their values are higher when the samples are more similar to each other. Therefore, in case of using Pearson correlation for these similarity measures, we substitute $d_{C}$ with $1-\rho_{C}$ for cell lines and $d_{D}$ with $1-\rho_{D}$ for drugs, where ρ denotes the Pearson correlation. Consequently, we compute the drug response by:

$$\;R^{*}\left(c^{*},d^{*}\right)=\frac{\sum_{d\in C_{d^{*}}}\sum_{c\in C_{c^{*}}}w_{C}\left(c,c^{*}\right)w_{D}\left(d,d^{*}\right)r\left(c,d\right)}{\sum_{d\in C_{d^{*}}}\sum_{c\in C_{c^{*}}}w_{C}\left(c,c^{*}\right)w_{D}\left(d,d^{*}\right)}.$$

The decay parameters $\zeta =\left(\alpha ,\beta \right)$ can be optimized on the training set by minimizing the error of response prediction as follows:

$$\;\zeta^{*}={\mathrm{argmin}}_{\zeta }\sum_{\left(c,d\right)\in \Gamma }{\left(r^{*}\left(c,d\right)-r\left(c,d\right)\right)}^{2},$$

where $r^{*}\left(c,d\right)$ is the prediction of drug response $r\left(c,d\right)$ in the training set.
