# Supplementary material for: Pan-Cancer Prediction of Cell-Line Drug Sensitivity Using Network-Based Methods
Source: Int J Mol Sci. 2022 Jan 19;23(3):1074. doi: 10.3390/ijms23031074 (PMC8835038; doi:10.3390/ijms23031074)
Supplement: Supplementary file 1 [file ijms-23-01074-s001.zip › Supplementary Figure S2.pdf]

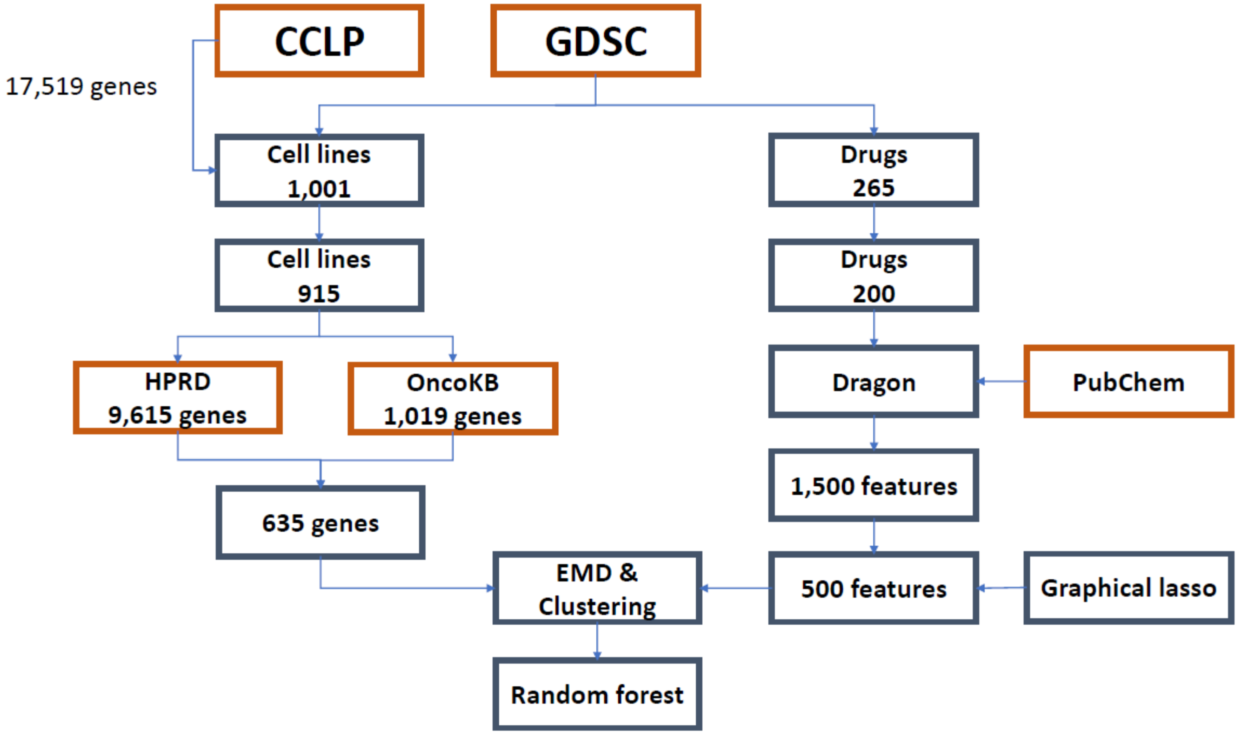

(A)

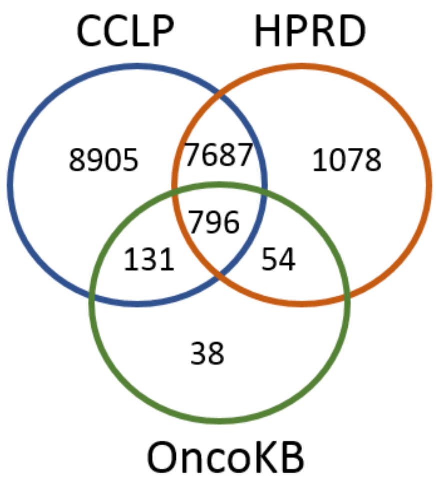

(B)

Figure S2. (A) Overview of data analysis; (B) The number of common genes among the gene sets in CCLP, HPRD, and OncoKB databases.
