# Supplementary material for: Pan-Cancer Prediction of Cell-Line Drug Sensitivity Using Network-Based Methods
Source: Int J Mol Sci. 2022 Jan 19;23(3):1074. doi: 10.3390/ijms23031074 (PMC8835038; doi:10.3390/ijms23031074)
Supplement: Supplementary file 1 [file ijms-23-01074-s001.zip › Supplementary Figure S3.pdf]

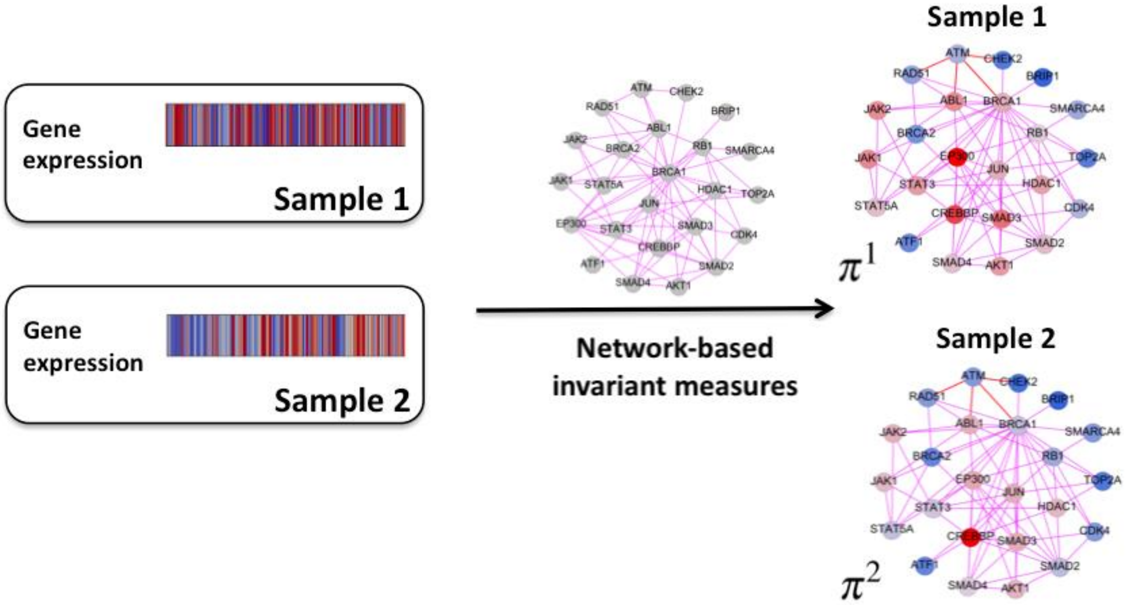

Figure S3. The invariant measures define a weighted network for each sample. The Wasserstein distance (EMD) calculates the most efficient way to move the distribution of invariant measures from one sample to another sample, where the cost is the shortest path in the network. Here, we show a small network for the purpose of illustration.
