# Supplementary material for: Pan-Cancer Prediction of Cell-Line Drug Sensitivity Using Network-Based Methods
Source: Int J Mol Sci. 2022 Jan 19;23(3):1074. doi: 10.3390/ijms23031074 (PMC8835038; doi:10.3390/ijms23031074)
Supplement: Supplementary file 1 [file Supplementary Figure S1.pdf › Supplementary Figure S1.pdf]

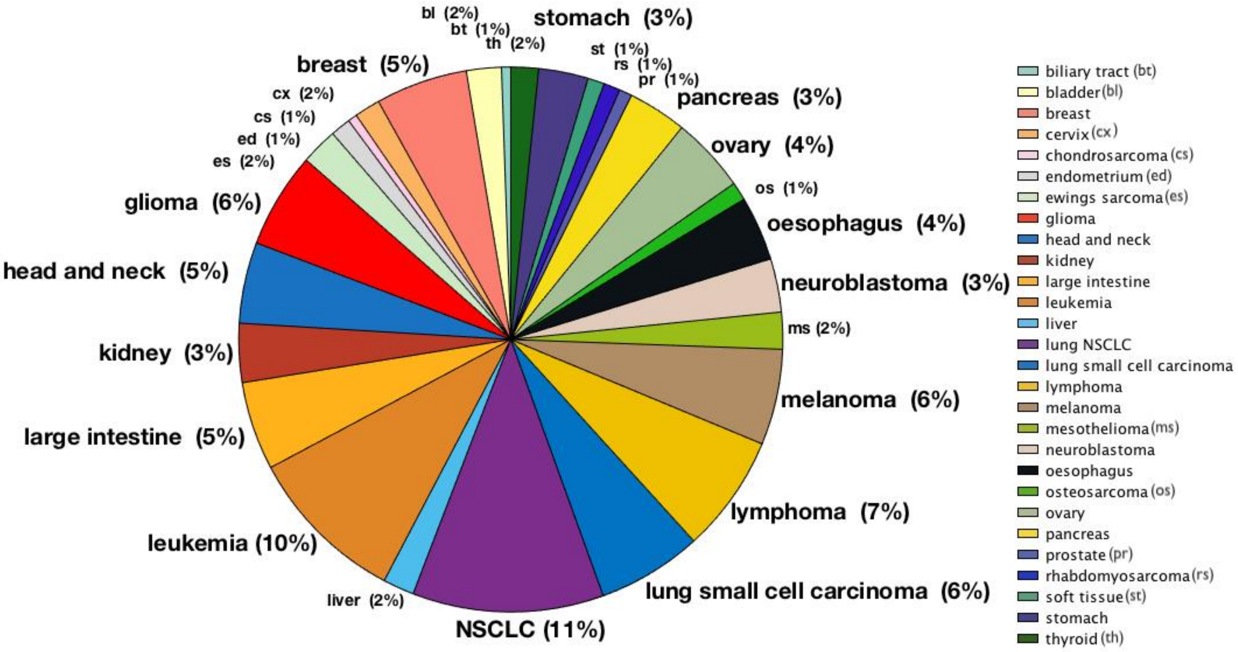

Figure S1. Distribution of the 915 cell lines from the GDSC dataset with respect to their cancer types. The largest cancer types are non-small cell lung cancer (NSCLC) (11%) and leukemia (10%). Lymphoma (7%), glioma (6%), melanoma (6%), and lung small cell carcinoma (6%) are the next major types.
